# Mild behavioural impairment-apathy and core Alzheimer's disease cerebrospinal fluid biomarkers

**DOI:** 10.1093/brain/awaf194

**Published:** 2025-06-02

**Authors:** Daniella Vellone, Rebeca Leon, Zahra Goodarzi, Nils D Forkert, Eric E Smith, Zahinoor Ismail

**Affiliations:** Faculty of Graduate Studies, University of Calgary, Calgary, Alberta T2N 1N4, Canada; Hotchkiss Brain Institute, Cumming School of Medicine, University of Calgary, Calgary, Alberta T2N 4N1, Canada; Hotchkiss Brain Institute, Cumming School of Medicine, University of Calgary, Calgary, Alberta T2N 4N1, Canada; Faculty of Graduate Studies, University of Calgary, Calgary, Alberta T2N 1N4, Canada; Mathison Centre for Mental Health Research and Education, Cumming School of Medicine, University of Calgary, Calgary, Alberta T2N 4Z6, Canada; Department of Community Health Sciences, Cumming School of Medicine, University of Calgary, Calgary, Alberta T2N 4N1, Canada; Department of Medicine, Cumming School of Medicine, University of Calgary, Calgary, Alberta T2N 4N1, Canada; O’Brien Institute for Public Health, Cumming School of Medicine, University of Calgary, Calgary, Alberta T2N 4Z6, Canada; Hotchkiss Brain Institute, Cumming School of Medicine, University of Calgary, Calgary, Alberta T2N 4N1, Canada; Department of Clinical Neurosciences, Cumming School of Medicine, University of Calgary, Calgary, Alberta T2N 4N1, Canada; Department of Radiology, Cumming School of Medicine, University of Calgary, Calgary, Alberta T2N 4N1, Canada; Alberta Children’s Hospital Research Institute, University of Calgary, Calgary, Alberta T3B 6A8, Canada; Hotchkiss Brain Institute, Cumming School of Medicine, University of Calgary, Calgary, Alberta T2N 4N1, Canada; Department of Community Health Sciences, Cumming School of Medicine, University of Calgary, Calgary, Alberta T2N 4N1, Canada; Department of Clinical Neurosciences, Cumming School of Medicine, University of Calgary, Calgary, Alberta T2N 4N1, Canada; Hotchkiss Brain Institute, Cumming School of Medicine, University of Calgary, Calgary, Alberta T2N 4N1, Canada; Mathison Centre for Mental Health Research and Education, Cumming School of Medicine, University of Calgary, Calgary, Alberta T2N 4Z6, Canada; Department of Community Health Sciences, Cumming School of Medicine, University of Calgary, Calgary, Alberta T2N 4N1, Canada; O’Brien Institute for Public Health, Cumming School of Medicine, University of Calgary, Calgary, Alberta T2N 4Z6, Canada; Department of Clinical Neurosciences, Cumming School of Medicine, University of Calgary, Calgary, Alberta T2N 4N1, Canada; Department of Psychiatry, Cumming School of Medicine, University of Calgary, Calgary, Alberta T2N 4N1, Canada; Department of Pathology and Laboratory Medicine, University of Calgary, Calgary, Alberta T2N 4N1, Canada; Faculty of Health and Life Sciences, Clinical and Biomedical Sciences, University of Exeter, Exeter EX1 2HZ, UK

**Keywords:** Core biomarkers, Amyloid Tau Neurodegeneration (ATN), Preclinical Alzheimer's disease, Prodromal Alzheimer's disease, Motivational deficits

## Abstract

Apathy is a common neuropsychiatric symptom (NPS) in Alzheimer's disease (AD) but can emerge earlier in prodromal and even preclinical stages as part of mild behavioural impairment (MBI-apathy), a syndrome defined by emergent and persistent NPS. In dementia, apathy is associated with higher morbidity, mortality and caregiver distress. However, the significance of MBI-apathy in dementia-free persons, including its associations with AD biomarkers, remains unclear. This study aimed to determine whether MBI-apathy is associated with biomarker evidence of amyloid-beta (Aβ) and tau [phosphorylated (p-tau) and total (t-tau)] in CSF. Because MBI predicts incident dementia better than NPS without MBI, we aimed to determine the association between apathy and AD biomarkers when it occurred as part of the MBI syndrome and when it did not.

Dementia-free participants with mild cognitive impairment or normal cognition from the Alzheimer's Disease Neuroimaging Initiative were stratified by NPS status (MBI-apathy, non-apathy MBI, non-MBI NPS and no-NPS) based on the Neuropsychiatric Inventory (NPI) or NPI-Questionnaire (NPI-Q). Linear regressions modelled cross-sectional associations between NPS status (predictor) and CSF biomarker ratios (Aβ_42_/Aβ_40_, p-tau_181_/Aβ_42_ and t-tau/Aβ42; primary outcomes) and levels (Aβ_40_, Aβ_42_, p-tau_181_ and t-tau; exploratory outcomes), adjusting for age, sex, apolipoprotein E4, education, Mini-Mental State Examination and NPI version. Hierarchical linear mixed-effects (LME) models assessed longitudinal associations over 2 years, incorporating random intercepts and slopes to account for repeated measures. Fixed effects included NPS status, all covariates from the linear regression model, and an interaction term between NPS status and time.

Among 477 participants (176 cognitively normal), 52 had MBI-apathy. Primary cross-sectional analyses showed that, compared with the no-NPS group, MBI-apathy was associated with higher CSF p-tau_181_/Aβ_42_ [11.25% (2.56%–20.68%); adjusted *P* = 0.018] and t-tau_181_/Aβ_42_ [10.26% (2.42%–18.70%); adjusted *P* = 0.018]. Exploratory analyses revealed that MBI-apathy was associated with higher CSF p-tau_181_ [5.98% (0.50%–11.77%); *P* = 0.032]. Primary LME models showed that MBI-apathy was associated with higher CSF p-tau_181_/Aβ_42_ [11.34% (2.55%–20.88%); adjusted *P* = 0.022] and t-tau_181_/Aβ_42_ [10.34% (2.41–18.88%); adjusted *P* = 0.022] over 2 years. Exploratory LME models revealed that MBI-apathy was associated with higher CSF p-tau_181_ [6.03% (0.56%–11.81%); *P* = 0.032] and t-tau [4.96% (0.07%–10.09%); *P* = 0.049] over 2 years.

MBI-apathy was significantly associated with core AD biomarkers cross-sectionally and longitudinally, over 2 years, underscoring its relevance as a marker of AD pathological burden. An overall MBI composite score might reflect a broader spectrum of pathology and warrants further investigation.

## Teaser

Vellone *et al.* studied 477 older adults and found that later-life emergent apathy is associated with key Alzheimer’s disease biomarker ratios in cerebrospinal fluid. Apathy may be a potential early warning sign of Alzheimer’s disease prior to the development of dementia.

## Introduction

Globally, there are >55 million people currently living with dementia, also known as major neurocognitive disorder, a condition characterized by substantial cognitive impairment and behavioural changes that are severe enough to compromise independence and interfere with daily functioning.^1^ Age is the most significant risk factor,^2^ and with the proportion of people aged ≥65 years continually rising, the number of individuals living with dementia is expected to increase in future decades. In 2030, prevalence is expected to reach 74.7 million,^3^ with the majority of dementia patients expected to have Alzheimer's disease (AD).

AD is characterized by the accumulation of abnormal proteins, including amyloid beta (Aβ) and phosphorylated tau (p-tau), in addition to total tau (t-tau), a marker of neurodegeneration in the brain.^4^ Recently, disease-modifying therapies have been approved for treatment of early AD, with demonstrated efficacy in reducing amyloid burden.^5^ With AD being the most frequent form of dementia and the most common neurodegenerative disease worldwide,^6^ these therapies provide hope for patients and clinicians, with potential public health implications.^7^

Although AD dementia is conceptualized primarily as progressive cognitive impairment (e.g. in memory, executive function, language) and functional decline, neurobehavioural changes (e.g. depression, anxiety, apathy) are exceedingly common.^8^ Cognitive [mild cognitive impairment (MCI) also known as minor neurocognitive disorder] and behavioural changes [mild behavioural impairment (MBI)] typically emerge before the functional decline characteristic of dementia. MCI is defined as noticeable cognitive decline that is not severe enough to interfere significantly with daily function.^8^ Later-life emergent and persistent neuropsychiatric symptoms (NPS) that may precede or accompany MCI represent MBI.^9^ Domains that make up MBI include apathy, affective dysregulation, impulse dyscontrol, social inappropriateness and psychosis.

Apathy is one of the most prevalent NPS and is characterized by diminished interest, initiative and emotional reactivity independent of primary motor or sensory deficits.^10-13^ These three dimensions align with recognized domains of apathy: diminished interest reflects the cognitive domain, involving reduced enthusiasm about usual activities; reduced initiative represents the behavioural domain, encompassing a lack of self-directed actions or motivation to initiate activities; and blunted emotional reactivity corresponds to the emotional domain, marked by a diminished capacity to express emotions.^11-14^ Given its frequent co-occurrence with cognitive and mood disorders, it is essential to distinguish apathy as a separate entity, with its own neurobiological underpinnings.^13^ This distinction is particularly relevant in the context of MBI where persistent and late-life-emergent apathy fulfils MBI criteria. MBI-apathy is a prominent MBI domain, which has been linked to greater risk for incident dementia, suggesting its potential utility as an early prognosticator for cognitive decline.^15^ Although MBI-apathy demonstrates greater incidence of clinically diagnosed AD dementia in comparison to those without NPS, associations with the underlying proteinopathies that serve as the hallmarks of AD are unknown. Investigating potential associations could help to determine whether MBI-apathy can be used as a proxy marker or early warning sign for early-stage AD. This knowledge could then inform screening practices in older adults and help to identify individuals requiring further evaluation, ultimately reducing diagnostic delays and improving healthcare resource allocation.

In this work, we investigate the association of MBI-apathy with core AD CSF biomarker ratios, Aβ_42/40_, p-tau_181_/Aβ_42_ and t-tau_181_/Aβ_42_, as our primary analyses, given that these ratios are more sensitive and accurate than individual biomarkers in reflecting AD pathophysiology.^16-18^ In addition, we conduct exploratory analyses to examine the relationships between MBI-apathy and individual biomarkers, Aβ_40_, Aβ_42_, p-tau_181_ and t-tau, to explore potential associations further. We hypothesized that in dementia-free individuals, MBI-apathy would be associated with a lower CSF Aβ_42/40_ ratio and with higher CSF p-tau_181_/Aβ_42_ and t-tau_181_/Aβ_42_ ratios, both cross-sectionally and longitudinally. Lower CSF Aβ and higher CSF tau levels are the typical fingerprint characteristic of AD, reflecting the accumulation of Aβ plaques, neurofibrillary tangles and neurodegeneration. By establishing a link between MBI-apathy and core AD biomarker ratios, our study aims to investigate the role of apathy as an early behavioural indicator of AD, potentially aiding in the early identification and intervention of individuals at risk.

## Materials and methods

### Study population: Alzheimer's Disease Neuroimaging Initiative

Data were drawn from the Alzheimer's Disease Neuroimaging Initiative (ADNI; https://adni.loni.usc.edu/). ADNI was launched in 2003 and is led by Principal Investigator Michael W. Weiner, MD. It is a privately and publicly funded multicentre study across North America and was established to develop standardized clinical, imaging, genetic and biomarker procedures in individuals along the cognitive spectrum ranging from normal to mild AD. This longitudinal dataset comprises the collection of demographics, diagnoses and neurological, genetic and neuropathological examination results collected half-yearly or yearly. All ADNI studies comply with Good Clinical Practice guidelines, the Declaration of Helsinki, and US regulations (21 CFR Part 50: Protection of Human Subjects and Part 56: Institutional Review Boards). ADNI protocol received approval from Institutional Review Boards at each participating site. Written informed consent was obtained from all participants prior to the initiation of any protocol-specific procedures.

### Participant selection

**Figure 1:**
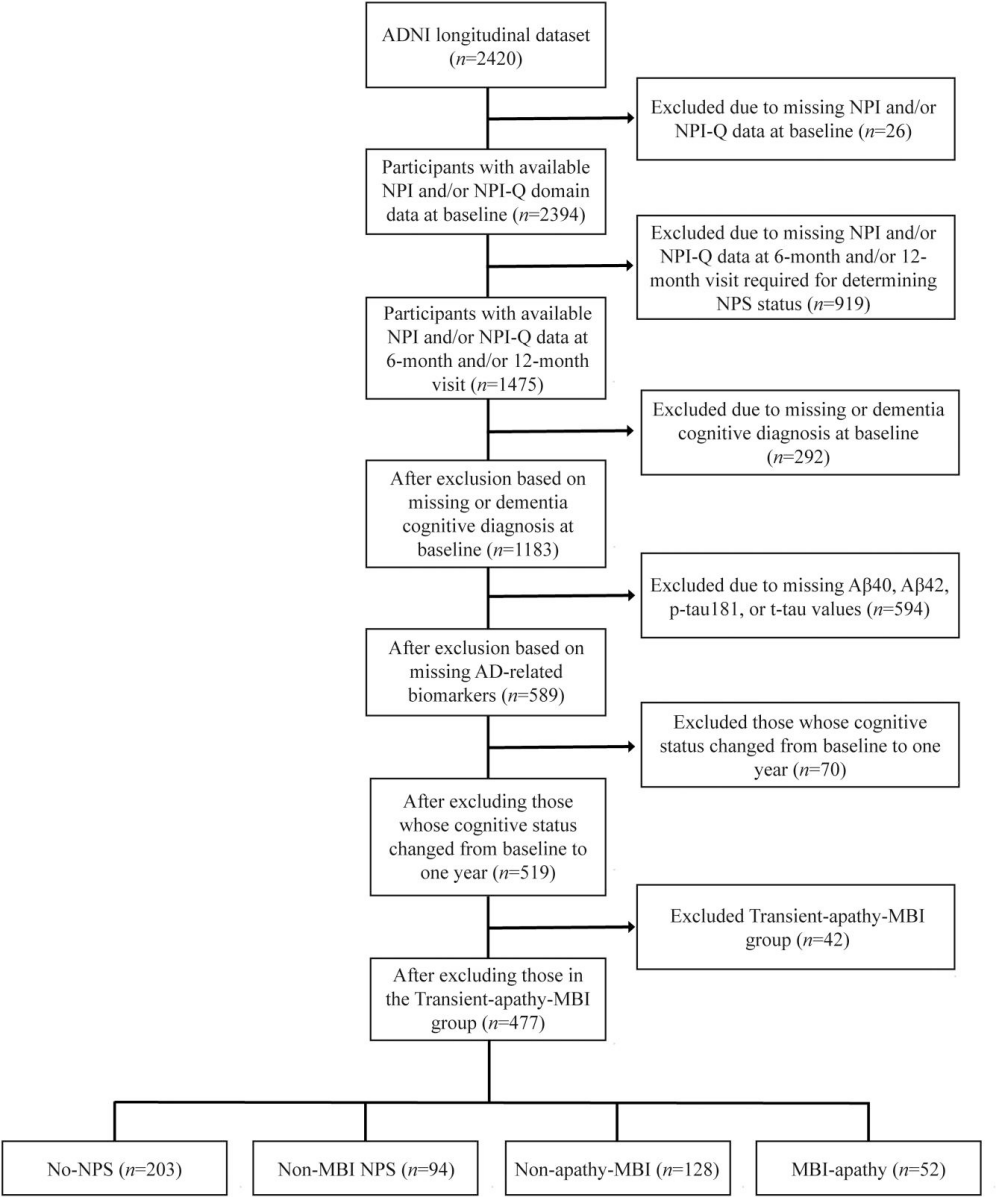
**Flow chart of participants from ADNI included for analysis.** ADNI = Alzheimer's Disease Neuroimaging Initiative; CN = cognitively normal; MBI = mild behavioural impairment; MCI = mild cognitive impairment; NPI = neuropsychiatric inventory; NPI-Q = neuropsychiatric inventory questionnaire; NPS = neuropsychiatric symptoms.

Study flow for primary data analysis is shown in Fig. 1. All participants who were cognitively normal (CN) or diagnosed with MCI enrolled from October 2004 to August 2022 were considered in this study. Cognitive diagnosis was determined by Mini-Mental State Examination (MMSE) and Clinical Dementia Rating (CDR). Those classified as CN had MMSE scores between 24 and 30, and a CDR score of 0. Participants with MMSE scores between 24 and 30, a CDR score of 0.5, memory complaints and objective memory loss as per education-adjusted scores on the Wechsler Memory Scale Logical Memory II, and who presented with preserved activities of daily living, were classified as having MCI.

Participants were included in this study if they had either complete Neuropsychiatric Inventory (NPI) (from ADNI-2, 3) or Neuropsychiatric Inventory Questionnaire (NPI-Q)^19^ (from ADNI-1, GO, 2, 3) domain scores necessary for determining NPS status, and at least two study visits within their first year to determine symptom persistence. To determine MBI status, NPI and NPI-Q scores were transformed into MBI domain scores using a published and validated algorithm.^20^ In this study, we operationalized MBI-apathy using the NPI and NPI-Q apathy domain scores. The NPI assesses apathy primarily through questions about loss of interest, lack of motivation and reduced emotional responsiveness, structured in a way that a severity score is obtained only if the initial screening question is endorsed. It is important to note that the NPI-Q assesses apathy using a single question that distills key aspects of the NPI apathy screening item, focusing on diminished interest in both personal activities and social engagement. Although this operationalization might not capture the full breadth of the MBI-apathy construct, it ensures consistency with widely used clinical tools and previous research. The presence of apathy was determined by an apathy domain score of >0. To meet the MBI-apathy symptom persistence criterion, apathy needed to be identified in at least two of three visits within the first year, at 0 and 6 months, 0 and 12 months, 6 and 12 months or 0, 6 and 12 months, as per the algorithm. Individuals were included in the MBI-apathy group irrespective of whether they had concurrent NPS in other MBI domains.

A non-apathy MBI four-domain score was obtained from the NPI- and NPI-Q-derived affect, impulse dyscontrol, social inappropriateness and psychosis domain scores. To meet the MBI four-domain symptom persistence criterion, affective dysregulation, impulse dyscontrol, social inappropriateness and/or psychosis domain scores had to be >0 in at least two of three visits within the first year, as per the algorithm, but with no apathy at these visits. The non-apathy MBI group was selected to determine the contributions of the other MBI domains as a composite risk group without the influence of apathy.

If NPS in any of the five MBI domains (apathy, affective dysregulation, impulse dyscontrol, social inappropriateness and psychosis) were present in only one of the three visits within the first year, participants were included in the non-MBI NPS group (owing to not meeting the MBI symptom persistence criterion).

The no-NPS group included participants without any NPS (MBI total score = 0) at all first-year visits.

Additionally, participants required a complete CSF biomarker profile (Aβ_40_, Aβ_42_, p-tau_181_ and t-tau) at baseline and 24 months for study inclusion. ADNI CSF concentrations of Aβ_40_, Aβ_42_, p-tau_181_ and t-tau were measured using Elecsys CSF immunoassays on a cobas e 601 analyser at the University of Pennsylvania.^21^

### Statistical analysis

Baseline demographic characteristics included age, sex and years of education. Clinical data included MMSE score and NPI/NPI-Q. Biomarkers included apolipoprotein E4 (*APOE4*) carrier status, CSF Aβ_42_/Aβ_40_, p-tau_181_/Aβ_42_ and t-tau/Aβ_42_ ratios, in addition to Aβ_40_, Aβ_42_, p-tau_181_ and t-tau levels. The MBI-apathy and non-MBI NPS group were compared against the no-NPS group using χ^2^ tests for categorical variables and one-way ANOVAs for continuous variables.

Both cross-sectional and longitudinal associations of NPS status with core AD biomarkers were modelled. Primary analyses focused on the CSF biomarker ratios (Aβ_42_/Aβ_40_, p-tau_181_/Aβ_42_ and t-tau/Aβ_42_), given their superior sensitivity and accuracy in reflecting AD pathophysiology, and exploratory analyses examined individual biomarkers (Aβ_40_, Aβ_42_, p-tau_181_ and t-tau). Linear regressions were used to model the cross-sectional association of NPS status (predictor) with Aβ_42_/Aβ_40_, p-tau_181_/Aβ_42_, t-tau/Aβ_42_, Aβ_40_, Aβ_42_, p-tau_181_ and t-tau CSF ratios and levels (continuous outcome variables), adjusting for age, sex, years of education, *APOE4* carrier status, MMSE score, neuropsychiatric assessment type (NPI, NPI-Q, combination) and number of visits used to determine NPS status (two or three visits). NPS status, *APOE4* carrier status, neuropsychiatric assessment type and the number of visits used to determine NPS status were all coded using dummy variables. NPS status was defined using four levels, including MBI-apathy, non-apathy MBI, non-MBI NPS and no-NPS, with the no-NPS group serving as the reference. *APOE4* carrier status was categorized into two levels, whereby individuals with one or more *APOE4* alleles were classified as *APOE4* carriers and those with no *APOE4* alleles as non-*APOE4* carriers, with non-*APOE4* carriers serving as the reference group. Dummy variables for neuropsychiatric assessment type corresponded to NPI for all visits, NPI-Q for all visits or a combination of NPI and NPI-Q, with NPI for all visits as the reference. Lastly, dummy variables for the number of visits used to determine NPS status were coded as two visits or three visits, with two visits serving as the reference.

Logarithmic transformations were applied to CSF biomarker measures owing to skewness, and all CSF biomarkers were winsorized at the 5th and 95th percentiles to limit the influence of outliers without excluding any participant data, resulting in data distributions that met the assumptions of normality for regression analyses.

Hierarchical linear mixed-effects (LME) models were implemented to assess the longitudinal relationship between NPS status (predictor) and CSF ratios and levels of Aβ_42_/Aβ_40_, p-tau_181_/Aβ_42_, t-tau/Aβ_42_, Aβ_40_, Aβ_42_, p-tau_181_ and t-tau (outcome variables) over a span of 2 years. Repeated measures included CSF biomarker levels, and only baseline measures of the other variables were used. The participant identity was modelled with random intercepts and slopes to account for individual variability. Fixed effects included NPS status, age, sex, years of education, *APOE4* carrier status, MMSE score, neuropsychiatric assessment type (NPI, NPI-Q, combination) and visit number. Additionally, an interaction term between NPS status and visit number was included to determine whether the slope of change in biomarkers over time differed between the NPS groups and the no-NPS group.

All statistical analyses were conducted in R v.4.3.2 using the stats package for linear models, the lmer package for LMEs, and the emmeans package to evaluate trends between the groups over time. The significance level for all analyses was set at *P* < 0.05. To account for multiple testing and control the false discovery rate (FDR), we applied the Benjamini–Hochberg procedure to the *P*-values obtained from primary analyses. Assumptions for linear regression modelling were satisfied and assessed using diagnostic plots generated by the base plotting functions in R. The diagnostic plots included residuals versus fitted, normal Q-Q, scale-location, and residuals versus leverage, which helped to evaluate linearity, normality of errors, equal variance of errors and the presence of influential data points.

## Results

### Participant demographics and characteristics

**Figure 2:**
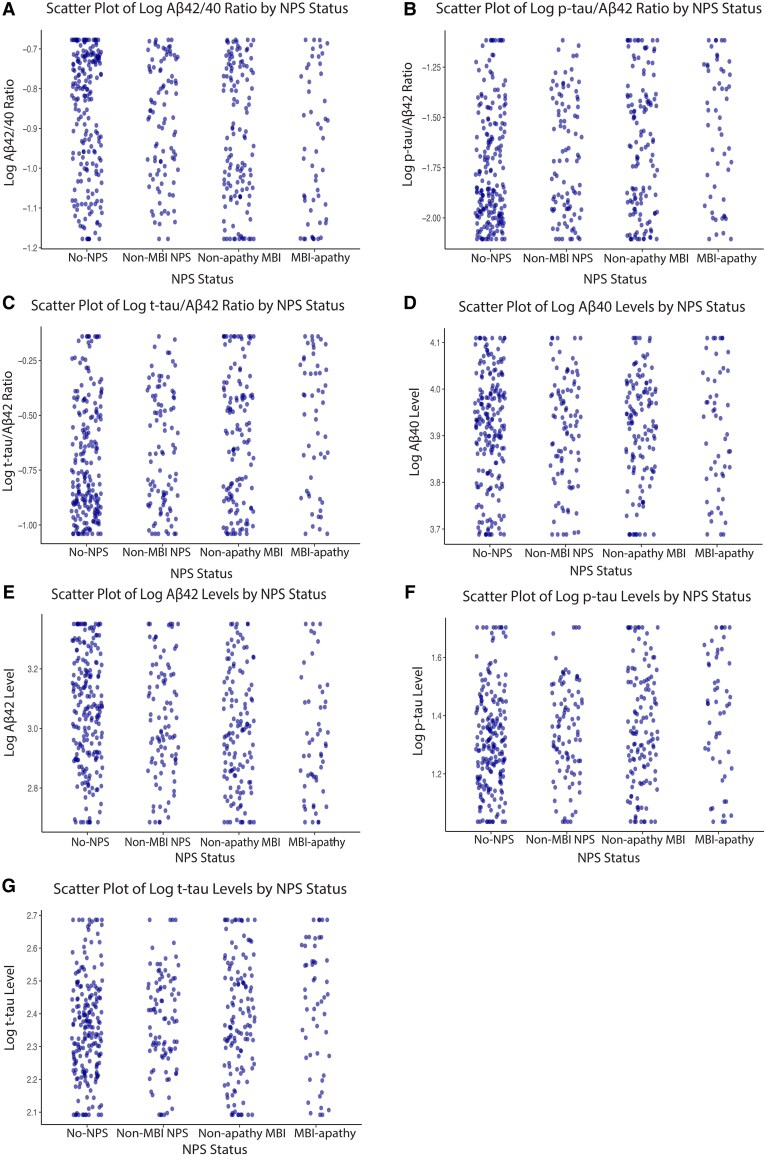
**Log-transformed baseline core CSF and Alzheimer’s disease biomarkers stratified by NPS status.** Scatter plots display log-transformed baseline values of: (**A**) Aβ_42_/Aβ_40_ ratio; (**B**) p-tau_181_/Aβ_42_ ratio; (**C**) t-tau/Aβ_42_ ratio; (**D**) Aβ_40_ levels; (**E**) Aβ_42_ levels; (**F**) p-tau_181_ levels; and (**G**) t-tau levels in CSF. Data points are stratified by NPS group: MBI-apathy, non-apathy MBI, non-MBI NPS and no-NPS (reference group). Aβ = amyloid-beta; MBI = mild behavioural impairment; NPS = neuropsychiatric symptoms; p-tau = phosphorylated tau; t-tau = total tau.

The sample comprised 203 participants with no NPS, 94 with non-MBI NPS, 128 with non-apathy MBI and 52 with MBI-apathy. Study participants were on average 72.5 years old, 47.4% were female and 63.1% had MCI, whereas 36.9% were cognitively normal. In comparison to the no-NPS group, participants with MBI-apathy were of similar age but more likely to be male, *APOE4* carriers and presented with a lower MMSE score (Table 1). The MBI-apathy group also had lower Aβ_42_ levels (*P* = 0.006) and higher p-tau_181_ (*P* = 0.01) and t-tau (*P* = 0.04) levels, a lower Aβ_42/40_ ratio (*P* = 0.002) and higher p-tau_181_/Aβ_42_ (*P* = 0.005) and t-tau/Aβ_42_ (*P* = 0.004) ratios compared with the no-NPS group (Table 1). Scatter plots for each of the core AD CSF biomarkers are shown in Fig. 2.

**Table 1:** Characteristics of participants

Factors		No-NPS (*n* = 203)	Non-MBI NPS (*n* = 94)	Non-apathy MBI (*n* = 128)	MBI-apathy (*n* = 52)	*P*-value
Age, years	Mean (SD)	72.7 (6.5)	73.5 (7.3)	70.9 (6.9)	72.7 (7.8)	**0.041** ^a^
	Median [Min, Max]	72.2 [56.3, 89.6]	74.0 [56.7, 88.3]	71.2 [55.5, 91.4]	74.4 [55.0, 88.6]	
Sex	Male	99 (48.8%)	43 (45.7%)	73 (57.0%)	35 (67.3%)	**0.021** ^b^
	Female	104 (51.2%)	51 (54.3%)	55 (43.0%)	16 (30.8%)	
Years of education	Mean (SD)	16.6 (2.6)	15.9 (2.8)	16.5 (2.4)	16.0 (2.6)	0.101^a^
	Median [Min, Max]	17.0 [12.0, 20.0]	16.0 [11.0, 20.0]	16.0 [10.0, 20.0]	16.0 [10.0, 20.0]	
*APOE4* carrier status	Non-APOE4 Carrier	136 (67.0%)	64 (68.1%)	72 (56.3%)	21 (40.4%)	**<0.001** ^b^
	APOE4 Carrier	67 (33.0%)	30 (31.9%)	56 (43.7%)	31 (59.6%)	
MMSE score	Mean (SD)	28.8 (1.47)	28.3 (1.69)	28.4 (1.59)	28.1 (1.58)	**0.006** ^a^
	Median [Min, Max]	29.0 [24.0, 30.0]	29.0 [24.0, 30.0]	29.0 [23.0, 30.0]	28.0 [24.0, 30.0]	
Aβ_42/40_	Mean (SD)	0.15 (0.05)	0.14 (0.05)	0.13 (0.05)	0.12 (0.05)	**0.002** ^a^
	Median [Min, Max]	0.15 [0.05, 0.24]	0.13 [0.06, 0.22]	0.12 [0.05, 0.23]	0.11 [0.04, 0.22]	
p-tau_181_/Aβ_42_	Mean (SD)	0.02 (0.02)	0.03 (0.02)	0.03 (0.03)	0.04 (0.04)	**<0.001** ^a^
	Median [Min, Max]	0.01 [0.00, 0.15]	0.02 [0.01, 0.09]	0.02 [0.01, 0.14]	0.03 [0.01, 0.24]	
t-tau/Aβ_42_	Mean (SD)	0.25 (0.21)	0.26 (0.17)	0.32 (0.25)	0.39 (0.32)	**<0.001** ^a^
	Median [Min, Max]	0.16 [0.06, 1.50]	0.21 [0.07, 0.81]	0.25 [0.07, 1.36]	0.35 [0.08, 2.10]	
Aβ_40_	Mean (SD)	8599.2 (2435.8)	8500.6 (2547.9)	8457.8 (2341.0)	8660.3 (2953.5)	0.942^a^
	Median [Min, Max]	8528.0 [3511.0, 18638.0]	8401.0 [2978.0, 19252.0]	8453.5 [2981.0, 15003.0]	8326.5 [4292.0, 15337.0]	
Aβ_42_	Mean (SD)	1260.2 (574.2)	1181.8 (567.2)	1088.8 (528.2)	1032.0 (572.3)	**0.012** ^a^
	Median [Min, Max]	1163.0 [231.0, 3132.0]	994.0 [317.0, 3278.0]	964.0 [300.0, 2893.0]	847.5 [366.0, 2981.0]	
p-tau_181_	Mean (SD)	23.1 (12.1)	23.9 (9.9)	25.5 (13.1)	29.3 (16.0)	**0.010** ^a^
	Median [Min, Max]	19.9 [8.3, 86.5]	21.8 [8.0, 64.5]	21.8 [8.2, 69.9]	27.6 [9.2, 92.1]	
t-tau	Mean (SD)	247.5 (109.2)	254.9 (96.2)	268.6 (118.5)	301.0 (142.0)	**0.018** ^a^
	Median [Min, Max]	227.6 [97.9, 811.7]	242.7 [81.5, 614.8]	237.2 [99.8, 642.7]	281.8 [106.2, 816.9]	

Significant *P*-values are shown in bold. Max = maximum; MBI = mild behavioural impairment; Min = minimum; MMSE = Mini Mental State Examination; NPS = neuropsychiatric symptoms; SD = standard deviation.

Abbreviations: Max = maximum; MBI = mild behavioural impairment; Min = minimum; MMSE = Mini Mental State Examination; NPS = neuropsychiatric symptoms; SD = standard deviation.

^a^One-way ANOVA.

^b^χ^2^ tests.

### Cross-sectional associations between MBI-apathy and core AD biomarkers

After adjusting for age, sex, years of education, *APOE4* carrier status, MMSE score, neuropsychiatric assessment type and number of visits, primary cross-sectional linear regression models revealed significant associations between MBI-apathy status and higher baseline p-tau_181_/Aβ_42_ and t-tau/Aβ_42_ ratios (Table 2). Exploratory analyses revealed a significant relationship between MBI-apathy status and higher baseline p-tau_181_ level. MBI-apathy status was not significantly associated with Aβ_42/40_, Aβ_40_, Aβ_42_ or t-tau. Comparatively, non-apathy MBI status was associated with lower Aβ_42/40_ and higher p-tau_181_/Aβ_42_ and t-tau/Aβ_42_ ratios, in addition to lower Aβ_42_, but no association was found for Aβ_40_, p-tau_181_ or t-tau. Non-MBI NPS status was not significantly associated with any of the core CSF AD biomarkers.

**Table 2:** Cross-sectional association between NPS group and CSF biomarkers in linear regression models adjusted for covariates

Outcome	Predictors	Percentage difference β	95% CI	*P*-value	FDR-adjusted *P*-value
Aβ_42/40^a^_	Non-MBI NPS	−1.57	−4.82 to 1.81	0.358	0.358
	Non-apathy MBI	−4.05	−6.94 to −1.06	**0.008**	**0.018**
	MBI-apathy	−4.06	−8.13 to 0.20	0.061	0.092
p-tau_181_/Aβ_42^a^_	Non-MBI NPS	4.06	−2.31 to 10.84	0.216	0.272
	Non-apathy MBI	7.97	1.95–14.35	**0.009**	**0.018**
	MBI-apathy	11.25	2.56–20.68	**0.010**	**0.018**
t-tau/Aβ_42^a^_	Non-MBI NPS	3.47	−2.29 to 9.57	0.242	0.272
	Non-apathy MBI	7.33	1.88–13.06	**0.008**	**0.018**
	MBI-apathy	10.26	2.42–18.70	**0.010**	**0.018**
Aβ_40^b^_	Non-MBI NPS	−0.75	−3.62 to 2.20	0.614	
	Non-apathy MBI	−0.02	−2.65 to 2.69	0.991	
	MBI-apathy	−0.19	−3.89 to 3.66	0.923	
Aβ_42^b^_	Non-MBI NPS	−2.59	−6.71 to 1.71	0.233	
	Non-apathy MBI	−4.62	−8.30 to −0.80	**0.018**	
	MBI-apathy	−4.26	−9.45–1.22	0.125	
p-tau_181^b^_	Non-MBI NPS	1.83	−2.28 to 6.12	0.387	
	Non-apathy MBI	3.15	−0.65 to 7.09	0.105	
	MBI-apathy	5.98	0.50 to 11.77	**0.032**	
t-tau^b^	Non-MBI NPS	1.16	−2.54 to 5.00	0.543	
	Non-apathy MBI	2.58	−0.83 to 6.11	0.140	
	MBI-apathy	4.75	−0.16 to 9.89	0.058	

Beta coefficients represent the estimated percent difference in the CSF marker compared to the No-NPS group at baseline. Models are adjusted for age, sex, years of education, *APOE4* carrier status, MMSE score, neuropsychiatric assessment type, and number of visits used to determine NPS status. Significant *P*-values are shown in bold. CI = confidence interval; FDR = false discovery rate; MBI = mild behavioural impairment; MMSE = Mini Mental State Examination; NPS = neuropsychiatric symptoms.

Abbreviations:

^a^Display primary results.

^b^Display exploratory results.

Non-apathy MBI and MBI-apathy did not differ significantly from one another in their associations with Aβ_42/_Aβ_40_ [percentage difference β (95% CI), −0.01% (−4.42% to 4.60%); *P* = 0.997], p-tau_181_/Aβ_42_ [3.04% (−5.32% to 12.13%); *P* = 0.487], t-tau/Aβ_42_ [2.73% (−4.85% to 10.93%); *P* = 0.490], Aβ_40_ [−0.17% (−4.02% to 3.83%); *P* = 0.932], Aβ_42_ [0.38% (−5.27% to 6.36%); *P* = 0.898], p-tau_181_ [2.75% (−2.77% to 8.59%); *P* = 0.335] or t-tau [2.11% (−2.85% to 7.33%); *P* = 0.410].

### Longitudinal associations between MBI-apathy and core AD biomarkers

**Figure 3:**
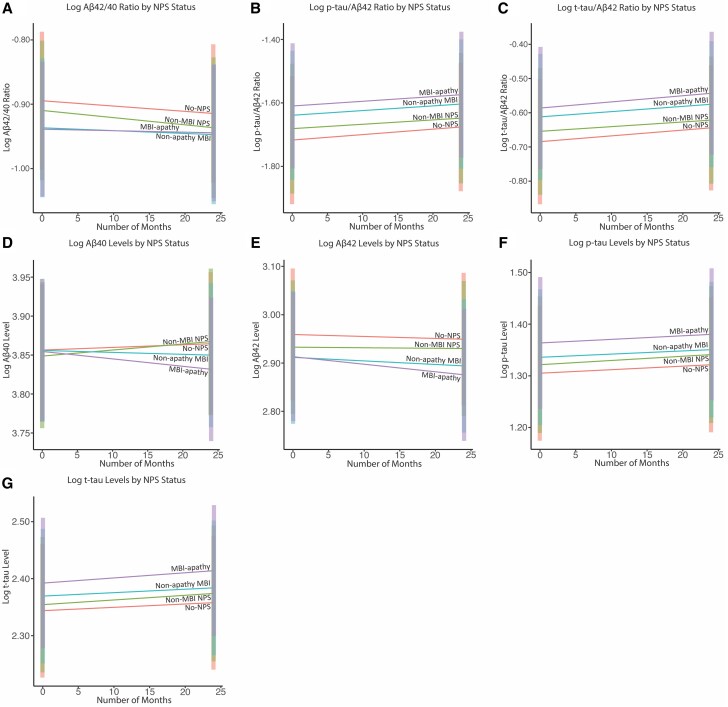
**Longitudinal trajectories of core CSF Alzheimer’s disease biomarkers over 2 years stratified by NPS status.** The estimated marginal mean trajectories and 95% confidence intervals for: (**A**) Aβ_42_/Aβ_40_ ratio; (**B**) p-tau_181_/Aβ_42_ ratio; (**C**) t-tau/Aβ_42_ ratio; (**D**) Aβ_40_ levels; (**E**) Aβ_42_ levels; (**F**) p-tau_181_ levels; and (**G**) t-tau levels in CSF over the 2-year follow-up period across groups defined by NPS status: MBI-apathy, non-apathy MBI, non-MBI NPS, and no-NPS (reference group). Aβ = amyloid-beta; MBI = mild behavioural impairment; NPS = neuropsychiatric symptoms; p-tau = phosphorylated tau; t-tau = total tau.

Longitudinal biomarker data for Aβ_40_ and Aβ_42_ were available in 158 of 477 participants at the 2-year mark, and data for p-tau_181_ and t-tau were available in 251 of 477 participants at the same time point. The 2-year biomarker evolution according to NPS status is shown in Table 3 and Fig. 3. Aβ_42/_Aβ_40_ [percentage change β (95% CI), −0.08% (−0.13% to −0.04%); *P* < 0.001], p-tau_181_/Aβ_42_ [0.17% (0.11%–0.23%)); *P* < 0.001], t-tau/Aβ_42_ [0.17% (0.11%–0.23%); *P* < 0.001], p-tau_181_ [0.07% (0.04%–0.10%); *P* < 0.001] and t-tau [0.06% (0.03%–0.09%); *P* < 0.001] changed significantly over time, whereas Aβ_40_ [0.03% (−0.02% to 0.09%); *P* = 0.216] and Aβ_42_ [−0.04% (−0.11% to 0.03%); *P* = 0.253] did not. Primary hierarchical LME analyses revealed that compared with no NPS, MBI-apathy was associated with higher p-tau_181_/Aβ_42_ and t-tau/Aβ_42_ ratios over 2 years. After correction for multiple comparisons, the association of MBI-apathy with the Aβ_42/_Aβ_40_ ratio was no longer significant, with an FDR-corrected *P*-value of 0.07. Exploratory analyses showed that MBI-apathy was also associated with higher p-tau and t-tau level over 2 years. Non-apathy MBI was associated with primary outcomes including lower Aβ_42_/Aβ_40_ ratio and higher p-tau_181_/Aβ_42_ and t-tau/Aβ_42_ ratios and with exploratory outcomes including lower Aβ_42_ level. Non-MBI NPS was not associated with any of the CSF biomarkers.

**Table 3:** Longitudinal association between NPS group and CSF biomarkers over 2 years modelled using linear mixed effect models adjusted for covariates

Outcome	Predictors	Percentage difference β	95% CI	*P*-value	FDR-adjusted *P*-value
Aβ_42/40^a^_	Non-MBI NPS	−1.50	−4.75 to 1.87	0.379	0.379
	Non-apathy MBI	−4.12	−7.01 to −1.15	**0.008**	**0.022**
	MBI-apathy	−4.33	−8.38 to −0.09	**0.048**	0.072
p-tau_181_/Aβ_42^a^_	Non-MBI NPS	3.66	−2.75 to 10.50	0.275	0.351
	Non-apathy MBI	8.13	2.03 to 14.60	**0.009**	**0.022**
	MBI-apathy	11.34	2.55–20.88	**0.012**	**0.022**
t-tau/Aβ_42^a^_	Non-MBI NPS	3.07	−2.73 to 9.21	0.312	0.351
	Non-apathy MBI	7.52	2.00–13.33	**0.008**	**0.022**
	MBI-apathy	10.34	2.41–18.88	**0.011**	**0.022**
Aβ_40^b^_	Non-MBI NPS	−0.77	−3.58 to 2.12	0.602	
	Non-apathy MBI	−0.05	−2.62 to 2.59	0.972	
	MBI-apathy	−0.18	−3.80 to 3.57	0.925	
Aβ_42^b^_	Non-MBI NPS	−2.59	−6.68 to 1.67	0.235	
	Non-apathy MBI	−4.62	−8.27 to −0.83	**0.019**	
	MBI-apathy	−4.50	−9.63 to 0.92	0.106	
p-tau_181^b^_	Non-MBI NPS	1.66	−2.44 to 5.94	0.437	
	Non-apathy MBI	3.13	−0.66 to 7.06	0.111	
	MBI-apathy	6.03	0.56–11.81	**0.032**	
t-tau^b^	Non-MBI NPS	1.07	−2.61 to 4.88	0.574	
	Non-apathy MBI	2.60	−0.80 to 6.11	0.140	
	MBI-apathy	4.96	0.07–10.09	**0.049**	

Beta coefficients represent the estimated percentage difference in the CSF marker in comparison to the No-NPS group across all time points. Except for Aβ_40_ and Aβ_42_, all biomarkers changed over time (not tabulated; see Fig. 3). Tests of interaction between NPS group and time were non-significant (*P* > 0.05), indicating that the slope of the biomarker change did not differ between NPS groups, with the exception that Aβ_40_ declined significantly over time in the MBI-apathy group [interaction β (95% CI), −0.13% (−0.25% to −0.01%), *P* = 0.036]. Models are adjusted for age, sex, years of education, *APOE4* carrier status, MMSE score, neuropsychiatric assessment type, number of months, and an interaction term between NPS status and number of months. Significant *P*-values are shown in bold. CI = confidence interval; FDR = false discovery rate; MBI = mild behavioural impairment; MMSE = Mini Mental State Examination; NPS = neuropsychiatric symptoms.

Abbreviations: CI = confidence interval; FDR = false discovery rate; MBI = mild behavioural impairment; MMSE = Mini Mental State Examination; NPS = neuropsychiatric symptoms.

^a^Display primary results.

^b^Display exploratory results.

Tests of interactions between NPS group and time were not statistically significant (*P* > 0.05), suggesting that, within the limits of our study, we did not detect differences in the slopes of biomarker changes between the groups. However, we acknowledge that the absence of a significant interaction does not suggest that that the slopes are identical. The only exception was Aβ_40_, which declined more rapidly in the MBI-apathy group [interaction β (95% CI), −0.13% (−0.25% to −0.01%), *P* = 0.036].

Non-apathy MBI and MBI-apathy did not differ significantly from one another in their associations with Aβ_42/_Aβ_40_ [−0.21% (−4.61% to 4.38%); *P* = 0.927], p-tau_181_/Aβ_42_ [2.96% (−5.48% to 12.16%); *P* = 0.508], t-tau/Aβ_42_ [2.62% (−5.04% to 10.90%); *P* = 0.518], Aβ_40_ [−0.13% (−3.89% to −3.78%); *P* = 0.947], Aβ_42_ [0.13% (−5.46% to 6.04%); *P* = 0.965], p-tau_181_ [2.81% (−2.70% to 8.64%); *P* = 0.329] or t-tau [2.31% (−2.64% to 7.51%); *P* = 0.373] over 2 years.

## Discussion

In this study of 477 dementia-free older adults, we explored the association between core AD CSF biomarkers and MBI-apathy. Associations were investigated, both cross-sectionally and longitudinally, over 2 years, in individuals with MBI-apathy, non-apathy-MBI and non-MBI-NPS relative to those with no-NPS. MBI-apathy was associated with higher p-tau_181_/Aβ_42_ and t-tau/Aβ_42_ cross-sectionally and longitudinally, over 2 years. Non-apathy-MBI was associated with lower Aβ_42/40_ and with higher p-tau_181_/Aβ_42_ and t-tau/Aβ_42_, cross-sectionally and longitudinally. Exploratory analyses revealed that MBI-apathy was associated with p-tau cross-sectionally and with p-tau and t-tau over 2 years. Exploratory analyses also revealed that non-apathy-MBI was associated with Aβ_42_ cross-sectionally and over 2 years. It is important to note that these exploratory analyses were not corrected for multiple comparisons; consequently, these findings should be interpreted with caution.

### Cross-sectional analysis

MBI-apathy was associated with higher p-tau_181_/Aβ_42_ and t-tau/Aβ_42_ ratios in comparison to those without NPS in our primary analyses, in addition to a higher p-tau_181_ level in our exploratory analyses. These findings indicate a greater burden of AD pathology and support the hypothesis that later-life emergent and persistent apathy might be linked to AD-related changes. Non-apathy MBI was associated with lower Aβ_42/40_ and with higher p-tau_181_/Aβ_42_ and t-tau/Aβ_42_ ratios in comparison to those without NPS. Exploratory analyses revealed that non-apathy MBI was associated with a lower Aβ_42_ level. The range of significant associations captured for most of the core AD biomarkers in the non-apathy MBI group appears reasonable, given that this group is composed of those who meet MBI criteria for affective dysregulation, impulse dyscontrol, social inappropriateness and/or psychosis. Our findings support the use of an overall MBI composite when implementing MBI for sample enrichment and early detection. The composite might be more sensitive to early AD pathological burden than a single domain, emphasizing the importance of assessing behavioural change across a broad spectrum.^22^ In comparison, non-MBI NPS showed no significant associations with core AD CSF biomarkers at baseline, suggesting that persistent symptoms (meeting MBI criteria) better align with underlying neuropathology than transient ones (not meeting MBI criteria).

Although some studies have reported statistically significant associations between Aβ_42_,^23-25^ p-tau^26^ and t-tau^26^ with apathy symptoms or severity across various cognitive statuses, most studies have not found associations for all core AD biomarkers. Notably, the study that reported associations with all core AD biomarkers (p-tau/Aβ_42_, t-tau/Aβ_42_ and Aβ_42_) did not adjust for cognitive status or cognitive scores, and the sample included only 11% with MCI.^23^ Other studies have found no relationship between apathy and core AD CSF biomarkers across the cognitive clinical spectrum.^27-29^

In the present study, the magnitudes of effect observed in the MBI-apathy group for the Aβ_42/40_ ratio and t-tau level were substantial and differed from those in the no-NPS group. However, the relatively small sample size in the MBI-apathy group are likely to have limited the precision of the estimates, which could have masked genuine associations. It is also possible, however, that these biomarkers do not exhibit associations with MBI-apathy in this sample. Future studies with larger cohorts and better MBI case ascertainment are needed to improve estimate precision and clarify these relationships.

Variations in findings across studies might stem from differences in study populations (e.g. cognitive status, disease stage), sample sizes and methodological approaches (e.g. adjustments for confounding variables). Importantly, the specific measures used to assess apathy and the definitions or cut-offs adopted might play a significant role. By failing to discriminate between persistent and transient groups and then analysing them together, the association and true signal for the group with persistent symptoms can become diluted.

A recent study exploring the utility of symptom persistence according to MBI criteria found associations with lower CSF Aβ_42/40_ and Aβ_42_, in addition to higher CSF p-tau_181_/Aβ_42_, t-tau_181_/Aβ_42_, p-tau_181_ and t-tau. In comparison, the transient NPS group had few associations.^30^ The same results were found for a study examining cross-sectional associations between MBI and plasma p-tau_181_.^31^ In our study, we did not find significant associations between non-apathy MBI status and p-tau_181_ and t-tau levels cross-sectionally. One possible explanation for this finding is that because the non-apathy MBI group (comprising individuals with MBI-affect, MBI-impulse dyscontrol, MBI-social inappropriateness and/or MBI-psychosis) was not associated with tau biomarkers, unlike the full MBI construct in our previous study,^30^ it might be that the MBI-apathy domain plays a contributing role in linking MBI to these biomarkers. Another possibility for this finding involves the timing and sequence of the emergence of pre-dementia NPS. Apathy tends to emerge later in the pre-dementia disease course, relative to affective symptoms and irritability/impulse dyscontrol symptoms,^32^ both of which occur more frequently pre-dementia than apathy.^33^ Furthermore, there might be slightly different effect sizes for biomarker associations with different MBI domains, dependent on the time course of domain emergence relative to the deposition of amyloid and the phosphorylation of tau. Thus, if affective symptoms emerge early, when amyloid is the primary pathological process, associations would be stronger with amyloid. Mid-stage domains, such as apathy, might emerge only after amyloid has already triggered tau phosphorylation, thus more strongly associating with tau. Consequently, the non-apathy MBI group, enriched with individuals exhibiting affective symptoms (which are more frequent and typically emerge earlier in the disease course than social inappropriateness and psychosis), might be more closely linked to amyloid pathology and contribute less to tau-related changes. Indeed, we have found MBI-affect to be associated with amyloid,^34^ supporting this hypothesis.

Furthermore, findings extend beyond CSF biomarkers to include amyloid- and tau-PET markers. MBI has been associated with higher Aβ PET uptake in regions that typically exhibit amyloidosis early during the course of AD,^35^ and significant associations with tau-PET have been observed in regions corresponding to Braak I–III pathological stages.^29,36^ Convergent findings across fluid and neuroimaging biomarkers underscore the robust associations between MBI and the core AD pathologies.

Accumulating evidence suggests that MBI might represent an early manifestation of AD, with dynamic changes in AD biomarkers contributing to the development and persistence of NPS. Consistent with inclusion of MBI in the National Institute on Aging Alzheimer's Association (NIA-AA) criteria for diagnosing and staging AD,^8^ our findings show that MBI-apathy is associated with established core 1 (Aβ proteinopathy, p-tau and secreted AD tau) and core 2 (AD tau proteinopathy) biomarkers, which reflect neuritic plaque and tau accumulation that are indicative of early- and later-stage AD pathology. Like the MBI-apathy group, the non-apathy MBI group showed higher p-tau_181_/Aβ_42_ and t-tau/Aβ_42_ ratios, while additionally showing lower Aβ_42/40_ ratios and Aβ_42_ levels in comparison to those without NPS. This finding suggests that although the MBI-apathy and non-apathy MBI groups both exhibit pathological features associated with AD, the MBI-apathy group appears to show more pronounced changes in biomarkers specifically associated with total tau pathology (i.e. neurodegeneration) and the non-apathy MBI group appears to display earlier changes primarily related to amyloid and tau phosphorylation. Given that MBI-apathy is associated with more advanced neurodegenerative changes, non-apathy MBI might distinguish itself from those without NPS earlier owing to its stronger associations with these early-changing core 1 biomarkers. The dynamic biomarker framework underscores that these early-changing core 1 biomarkers are crucial for detecting evolving neurodegenerative changes that are indicative of initial disease stages.^8^ Notably, the p-tau/Aβ_42_ ratio has been recognized as a strong predictor of cognitive decline and incident dementia,^37^ supporting the notion that MBI-related changes might reflect a greater burden of AD neuropathology. In contrast to the MBI-apathy and non-apathy MBI groups, which exhibited clear associations with distinct AD biomarker profiles, individuals with transient NPS did not show similar biomarker patterns. This lack of association suggests that transient NPS might not be linked directly to the same underlying pathological processes as those observed in MBI-apathy and non-apathy MBI. Instead, transient NPS might arise from a variety of other causes, such as situational factors or stressors.^9^

Rigorous criteria for identifying individuals with MBI are crucial for elucidating the relationships between neuropsychiatric changes and core AD biomarkers. Although the specific mechanisms remain to be determined, our study indicates that MBI-apathy and other NPS meeting MBI criteria are associated with AD pathology. Distinguishing MBI from transient NPS might enhance the identification of individuals at risk and improve understanding of NPS roles in preclinical and prodromal AD stages. Importantly, our findings underscore the clinical benefit of using assessment tools, such as the MBI-C, which captures symptom persistence over a 6-month period in a single visit. This approach minimizes patient burden while reliably distinguishing persistent symptoms from transient ones, the latter more likely to be linked to non-neurodegenerative aetiologies, ultimately facilitating early risk stratification and targeted intervention.

### Longitudinal analysis

MBI-apathy was associated with AD-related biomarker changes over a 2-year period. More specifically, MBI-apathy was associated with higher p-tau_181_/Aβ_42_ and t-tau/Aβ_42_ ratios over 2 years in the primary analyses, consistent with our hypotheses. MBI-apathy was also associated with greater p-tau_181_ and t-tau levels over 2 years in the exploratory analyses. Although our analysis did not reveal statistically significant differences in the slopes of biomarker change between the MBI-apathy group and the other NPS groups (with the exception of Aβ_40_), MBI-apathy status remained a significant predictor of overall longitudinal biomarker levels and ratios. It is important to note that the absence of a statistically significant interaction does not indicate conclusively that the rate of pathological progression is equivalent across groups, because this finding might be related to the limited statistical power or variability in our sample. Thus, research with larger cohorts is needed to determine whether differences in the rate of AD biomarker accumulation exist between these groups. The ADNI sample purposefully enriches for amyloid positivity, and it is possible that NPS-group specific trajectories diverge earlier in the course of disease. Nonetheless, consistent with our cross-sectional findings, non-apathy MBI status predicted lower Aβ_42/40_ and greater p-tau_181_/Aβ_42_ and t-tau/Aβ_42_ ratios according to our primary analyses, in addition to lower Aβ_42_ levels according to our exploratory analyses, in comparison to those with no NPS. In contrast to MBI-apathy and non-apathy MBI status, non-MBI NPS status was not significantly associated with core AD biomarkers over a 2-year period.

The finding that MBI-apathy is associated with changes in AD biomarkers over a 2-year period in CN and MCI individuals is novel. Most previous research has focused solely on baseline biomarker levels and apathy presence or severity rather than tracking biomarker trajectories over time. Past research suggests that lower baseline Aβ_42_ might predict greater incident apathy,^38,39^ whereas some studies have found no significant associations between CSF biomarker changes and the severity of apathy.^27^ The null findings might be attributable to methodological differences regarding apathy persistence, which our study explored purposefully. Thus, our findings build on these previous studies and extend the work to MBI-apathy. We demonstrated that MBI-apathy status at baseline is associated with greater tau pathology over 2 years among CN and MCI individuals. The observed biomarker profile aligns with other longitudinal studies showing that global MBI predicts AD-related CSF and plasma biomarker trajectories.^30,31^ We observed that MBI-apathy was associated with a higher Aβ_42/40_ ratio over a 2-year period. However, this association did not remain statistically significant after correction for multiple comparisons. Although the effect size suggests a potential difference between individuals with MBI-apathy and those with no-NPS, the lack of statistical significance might reflect insufficient power or a true absence of an association. Given the relatively small sample size of the MBI-apathy group, the precision of the estimates is limited. Future studies with larger sample sizes are needed to clarify whether these effects are robust or whether they reflect variability in biomarker progression.

The predictive value of baseline core AD biomarkers (p-tau_181_/Aβ_42_, t-tau/Aβ_42_, Aβ_42_, p-tau_181_ and t-tau) on NPS over time was examined previously, showing that levels of each biomarker were associated with increased NPI scores over time.^40^ Baseline MBI status has also been associated with lower Aβ_42/40_ and with higher p-tau_181_/Aβ_42_ and t-tau/Aβ_42_ ratios, in addition to lower Aβ_42_ and higher p-tau_181_ and t-tau levels in CSF over 4 years^30^ and higher plasma p-tau_181_ over the same time frame.^31^ Study concordance between MBI status and established AD biomarker profiles provides further evidence that persistent NPS meeting MBI criteria might represent behavioural manifestations of the AD pathological cascade at preclinical and prodromal stages.

Our findings also align with the broader MBI literature on cognitive decline and incident dementia,^41-53^ in addition to genetic,^54,55^ neuroimaging^22,56,57^ and fluid biomarkers.^31,34,58^ Survival analyses show that those with MBI-apathy are more likely to develop dementia earlier, with the majority progressing to AD dementia, suggesting that MBI-apathy might serve as an early marker of underlying AD pathology.^15^ Our findings reinforce the utility of incorporating symptom persistence, consistent with MBI criteria, into dementia prognostication to improve specificity of the group at risk. Our present study expands on prior research by revealing clear relationships between MBI-apathy and the complete range of CSF biomarkers outlined in the NIA-AA research framework for AD.^8^ Additionally, our findings for the non-apathy MBI group underscore the presence of early pathological changes associated with both amyloid and tau. The results indicate that non-apathy MBI reflects early-stage amyloid-related changes consistent with core 1 biomarkers, reinforcing the importance of incorporating MBI criteria globally to gain a better understanding and predict progression of AD dementia.

Together, our findings underscore a robust biological basis for apathy symptoms aligned with MBI criteria and highlight the utility of MBI-apathy and global MBI as a composite in identifying preclinical and prodromal stages of AD.

### Limitations

It is important to acknowledge several limitations of this study. First, ADNI is a restricted cohort with cognitive criteria that enrich participants for AD and minimize comorbid vascular disease, comparable to samples enrolled in AD clinical trials. Moreover, the relatively homogeneous nature of the sample along racial, ethnic, cultural and educational lines hinders the broader applicability of study findings. Additionally, although the no-NPS group served as the closest approximation to a control group, it included both CN individuals (60.6%) and those with MCI (39.4%). This grouping reflects the challenge of defining a completely asymptomatic control group in ageing populations, particularly when studying early NPS. Future studies should determine whether the significant MBI-apathy and AD biomarker associations found using the ADNI dataset can be replicated in the broader population. Second, given that CSF data were collected in only a subsample of ADNI, exploration of CSF biomarkers is somewhat limited. Furthermore, application of MBI criteria for apathy emergence and persistence also restricts the sample. Although identifying a higher-risk subsample out of a broader sample of persons with NPS is an *a priori* goal of the MBI criteria, the resulting smaller sample size can reduce statistical power in the types of analyses completed here. Moreover, our inability to analyse a transient-apathy group separately, owing to its small sample size (*n* = 42), is an additional limitation. Also, the definition of apathy in the NPI-Q is relatively brief, which might limit its sensitivity in capturing the full spectrum of apathy symptoms. However, prior research suggests that even a simple question assessing lack of interest can be predictive of underlying neurodegenerative pathology,^59^ indicating that despite its brevity, the NPI-Q apathy item might still capture meaningful clinical associations. Future studies could use the MBI-C, which identifies emergent and persistent NPS based on a 6-month time frame in a single assessment.^60^ The MBI-C has been validated in clinical and research populations^47,61-67^ and has been used in AD biomarker studies,^35,68,69^ thereby eliminating the need for multiple visits while reliably capturing symptom persistence.

Plasma biomarkers are increasingly gaining attention owing to scalability and accessibility. Although CSF biomarkers have historically had higher sensitivity and specificity for AD pathology,^8,17,70^ additional studies are required with plasma biomarkers and MBI to extend the work with CSF.

## Conclusion

Understanding the neurobiological associations between apathy, one of the most prevalent NPS, and AD, the most common type of dementia, is pivotal. Our findings suggest that apathy symptoms that meet MBI criteria might be linked to AD proteinopathies. Serving as a relatively early behavioural indicator, MBI-apathy can manifest prior to or concurrent with cognitive symptoms, reinforcing the importance of integrating behavioural assessments, such as apathy screening, alongside cognitive evaluations in clinical and research settings.^71^ Identifying and addressing apathetic behaviour when it emerges provides individuals with a proactive opportunity to recognize potential AD and seek timely medical guidance. Consulting a clinician about apathy not only facilitates early detection and better prognostication but also supports timely interventions, whether pharmacological or non-pharmacological, both of which might be more effective when administered early. Taking a proactive approach might also help patients and their families prepare better for a potential dementia diagnosis, thereby easing the burden on caregivers.

## Data Availability

Redistribution of ADNI data is prohibited as per the ADNI Data Sharing Publication Policy and Data Use Agreement (https://adni.loni.usc.edu/wp-content/uploads/how_to_apply/ADNI_DSP_Policy.pdf). Data from ADNI are available only by request and can be found here: https://adni.loni.usc.edu/. Authors can share the data cleaning script with interested parties.
